# HIV AND LUPUS ERYTHEMATOSUS: A DIAGNOSTIC DILEMMA

**DOI:** 10.4103/0019-5154.41652

**Published:** 2008

**Authors:** Feroze Kaliyadan

**Affiliations:** *From Department of Dermatology, Amrita Institute of Medical Sciences and Research Centre, Kochi, Kerala, India*

**Keywords:** *ANA*, *antinuclear antibody*, *HIV positivity*, *lupus erythematosus*

## Abstract

A 30-year-old female patient presented to us with erythema over the face and raised hyperpigmented, scaly skin lesions, mainly over photo-exposed parts, of about 5-month duration. Based on her clinical features and initial laboratory finding, we considered the possibility of a connective tissue disease. On further follow-up, she was found to be human immunodeficiency virus positive (confirmed by Western blot).

## Introduction

The diagnosis of a human immunodeficiency virus (HIV) infection in the setting of a connective tissue disorder like systemic lupus erythematosus (SLE) and vice versa have been vexing issues. It is well documented that HIV infection can mimic the presentation of autoimmune disorders, although there have been various conflicting reports on how exactly HIV infection affects the course and prognosis of diseases like SLE.^1^ We report a HIV positive case presenting with features simulating SLE.

## Case History

A 30-year-old female patient working as an insurance agent presented to us with relatively asymptomatic skin lesions mainly over the face and upper limbs of 5-month duration. The lesions had been gradually increasing in number over the last few months. She complained of exacerbation - increased erythema and burning sensation over the facial lesions on exposure to sunlight. Other than the skin lesions, she also complained of weight loss, arthralgia (without arthritis) and fatigue.

**Fig. 1:**
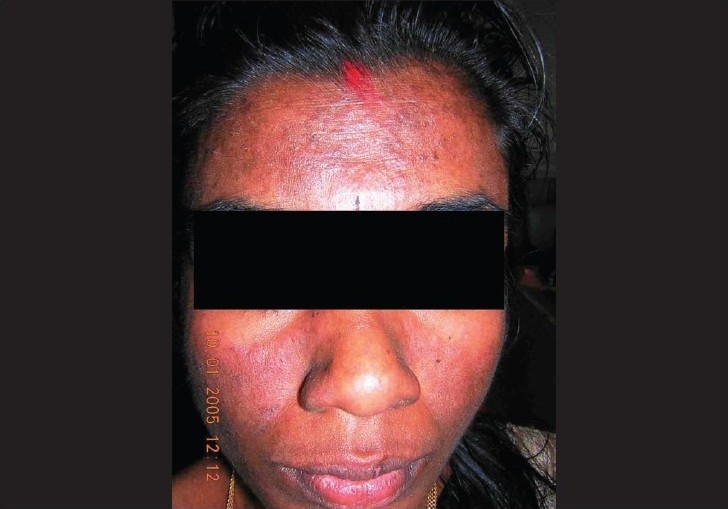
Malar erythema with minimal involvement of nasolabial fold

**Fig. 2:**
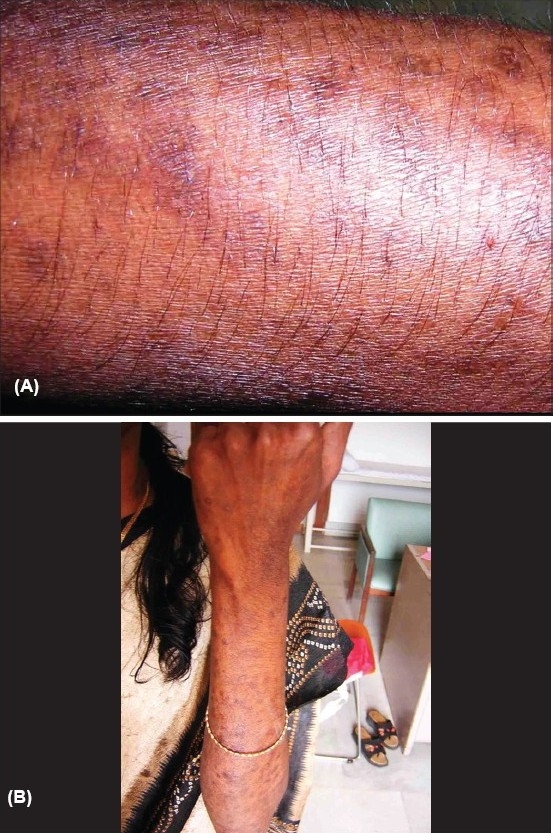
(A) Papular lesion with mild scaling mainly on photo-exposed areas. (B) Papular lesion with mild scaling mainly on photo-exposed areas

On examination, the skin lesions were distributed mainly over the face and upper limbs with a definite photoaccentuation (Figs. 1 and 2). The lesions were mainly hyperpigmented papules and plaques with mild scaling. There was no evidence of definite atrophy or telangiectasia. Her face showed an ill-defined malar erythema and slight scaling (Fig. 1). There was no evidence of any oral/nasopharyngeal ulcers.

We investigated the patient to rule out an underlying connective tissue disorder. Her ANA screen was positive with a significantly high titre. Other significant findings on investigation included an elevated erythrocyte sedimentation rate (ESR) and pancytopenia, with peripheral smear examination showing evidence suggestive of a haemolytic anaemia with reticulocytosis. Her hepatic and renal parameters were within normal limits. Her skin biopsy findings showed evidence of only a non-specific lymphocytic dermatitis, and immunofluorescence was also negative. Anti-double stranded (anti-DS) DNA titre was within normal limits; serological tests for syphilis were also negative. She was advised sunscreens and topical steroids, with which the lesions improved considerably. Around 4 weeks after her first visit, the patient presented to the Emergency Department with a history of fall and an episode of seizures. History wise the seizures appeared to be of the generalized tonic-clonic type. All pertinent investigations, including CT scan of the head and EEG, were found to be within normal limits. Within a short time, the patient developed a persistent productive cough and dysphagia. Examination revealed extensive crepitations in all lung fields and oropharyngeal candidiasis. The patient was admitted and blood was sent for HIV serology. HIV ELISA was positive and on further testing, Western blot was also found positive. Her CD4 count was 62/mm^3^. The patient's X-ray chest showed diffuse ill-defined infiltrates, a possibility of *Pneumocystis carinii pneumonia* was considered and the patient started on cotrimoxazole. In view of her low CD4 count and symptomatic disease, the patient was started on antiretroviral therapy (ART). Following ART, the patient's general condition improved and there had been no further recurrence of significant skin lesions.

## Discussion

The association between autoimmune diseases and retroviral infections has been studied extensively. It is well documented that the presentation of HIV infection can mimic that of autoimmune phenomena. Fevers, lymphadenopathy, rash, renal dysfunction, neurological and haematological disorders, sicca syndrome and polyarthralgias have been described.^2^ Photosensitivity is a frequently reported dermatological manifestation of HIV infection and is generally considered to be a manifestation of late HIV infection.^3^ Opportunistic infections like candidiasis have also been reported in SLE.^4^ Retroviral infections including HIV have been proposed as a possible etiological factor in autoimmune diseases such as SLE and Sjogren's syndrome.^5^,^6^ Antibodies to retroviral proteins have been demonstrated in patients with SLE.^5^ It is still uncertain whether the demonstration of autoimmunity in HIV positive patients has any bearing on the course or prognosis. There has been a reported case of lupus who was infected with HIV while on treatment.^7^ Stored sera had apparently shown the precise time of seroconversion and the authors noted clinical improvement as well as disappearance of autoantibodies following seroconversion. Some authors have even suggested that the presence of autoimmune diseases like SLE may in some way be protective for HIV.^8^

In our patient, virtually all her symptoms would have been consistent with both SLE and HIV infection. Photosensitivity, loss of weight, fatigue and seizures are well-known components of lupus, as are the findings of a strongly positive ANA and pancytopenia. Although the biopsy findings were negative, the patient satisfied four out of the eleven revised American College of Rheumatology (ACR) criteria required for the diagnosis of lupus erythematosus, namely photosensitivity, haematological disorder, antinuclear antibody and seizures (satisfying the definitions as per the ACR criteria). However, as we have discussed, all of the above can be explained on the basis of an underlying HIV infection. One of the problems in cases like ours would be the question of whether to start immunosuppressant medications under the presumption of autoimmune disease in a patient with advanced HIV.

While presenting this case, we would like to stress on the importance of doing a serological screening to rule out HIV infection in cases of suspected autoimmune disease. HIV has by now probably replaced syphilis as the “great mimicker” and we feel that it would be warranted to screen for HIV more often than is generally done now in the context of evaluation of suspected autoimmune disease. Similarly, it should be kept in mind that photosensitivity is a definite, well-documented feature of advanced HIV infection. Persistent and unexplained photosensitivity in patients with HIV risk factors or with suggestive laboratory finding should definitely warrant an HIV serology. In addition, the question of the validity of the diagnostic criteria of lupus in the setting of HIV positivity would probably require further refinement in future.
